# Supplementary material for: Ethanolamine-induced assembly of microcompartments is required for Fusobacterium nucleatum virulence
Source: mBio. 2024 Dec 23;16(2):e03405-24. doi: 10.1128/mbio.03405-24 (PMC11796344; doi:10.1128/mbio.03405-24)
Supplement: Supplemental material — Supplemental figures and tables. [file mbio.03405-24-s0001.pdf]

# Ethanolamine-induced assembly of microcompartments is required for *Fusobacterium nucleatum* virulence

**Dana S. Franklin[1], Yi-Wei Chen[2], Yimin Chen[2], Manuel Wittchen[3], Angela Agnew[4], Alexis Luu[2], Julian P Whitelegge[5], Z Hong Zhou[4], Andreas Tauch[4], Asis Das[6], and Hung Ton-That[1,2,4†]**

*[1]Molecular Biology Institute, University of California, Los Angeles, California, USA; [2]Division of Oral & Systemic Health Sciences, School of Dentistry, University of California, Los Angeles, California, USA; [3]Center for Biotechnology (CeBiTec), Bielefeld University, Bielefeld, Germany; [4]Department of Microbiology, Immunology & Molecular Genetics, University of California, Los Angeles, Los Angeles, CA, USA; [5]Pasarow Mass Spectrometry Laboratory, NPI-Semel Institute, University of California Los Angeles, Los Angeles, California, USA; [6]Department of Medicine, Neag Comprehensive Cancer Center, School of Medicine, University of Connecticut Health Center, Farmington, CT, USA*

[†]To whom correspondence may be address: Hung Ton-That, htonthat@dentistry.ucla.edu

Running Title: *BMC formation in fusobacterial virulence*

Keywords: *Fusobacterium nucleatum*, bacterial microcompartment, ethanolamine utilization, preterm birth, electron microscopy

## Supporting Figures

**Figure S1: Similarity of *F. nucleatum* $EutM_1$ and $EutM_2$. (A)** Sequence alignment of $EutM_1$ and $EutM_2$ was performed by CLUSTAL (1, 2). **(B)** 3-D structures of $EutM_1$ (green) and $EutM_2$ (coral) were predicted by AlphaFold (3). Superimposed images of $EutM_1$ and $EutM_2$, as well as *E. coli* EutM (PDB:3MPY; cyan) (4) and $EutM_2$, are shown.

**Figure S2: Expression of *eut* genes in *F. nucleatum* ATCC 25586 in response to ethanolamine.** *F. nucleatum* ATCC 25586 cells were grown at 37ºC in minimal media supplemented with 50 mM of EA for 8 h and harvested for total RNA extraction. Expression of indicated genes was determined by qRT-PCR, with 16S RNA as control. Error bars indicate means and standard deviations of three biological replicates.

**Figure S3: Quantification of BMCs. (A)** BMCs of the indicated strains, grown in the conditions as described in Fig. 3A, were isolated and analyzed by electron microscopy (EM) at a nominal magnification of 26,000 x. The number of BMCs at this magnification was quantified from representative images. Student's *t*-test was used for statistical analysis between the parent and other strains, with GraphPad Prism. Error bars indicate means and standard deviations of three biological replicates; **, $P<0.01$; ***, $P<0.001$. **(B)** BMCs of cells of the parent and indicated mutant strains grown in conditions and embedded as described in Fig. 4A-4F were analyzed by thin-section EM at a nominal magnification of 5,600 x. Number of BMCs was quantified from representative images at this magnification, and Student's t-test was used for statistical analysis between parent and individual mutant. Error bars indicate means and standard deviations of three biological replicates; **, $P<0.01$; ***, $P<0.001$. **(C)** BMCs of cells of the parent and indicated mutant strains, co-cultured with HTR-8/SVneo and embedded in resin as described in Fig. 5A-5F, were analyzed by thin-section EM at a nominal magnification of 5,600 x. Number of BMCs was quantified from representative images at this magnification, and Student's t-test was

used for statistical analysis between parent co-cultured with HTR-8/SVneo cells and individual experimental conditions. Error bars indicate means and standard deviations of three biological replicates; ***, P<0.001.

**Supporting Tables**

**Table S1: RNA-seq analysis of the parent and *eutV* mutant strains grown in rich media**

**Table S2: Mass spectrometry analysis of isolated BMCs**

**Table S3: Bacterial strains and plasmids used in this study**

| Strains & Plasmids | Description | Reference |
|---|---|---|
| *Strain* | | |
| *F. nucleatum* ATCC 23726 | Type strain | (5) |
| *F. nucleatum* CW1 | Derivative of 23726; lacking *galK* | (5) |
| *F. nucleatum* Δ*eutN* | Isogenic derivative of CW1 lacking *eutN* | This study |
| *F. nucleatum* Δ*eutL*/Δ*eutM*$_1$/Δ*eutM*$_2$ | Isogenic derivative of CW1 lacking *eutL*, *eutM*$_1$, and *eutM*$_2$ | This study |
| *F. nucleatum* Δ*eutL*/Δ*eutM*$_1$/Δ*eutM*$_2$/Δ*eutN* | Isogenic derivative of CW1 lacking *eutL*, *eutM*$_1$, *eutM*$_2$, and *eutN* | This study |
| *F. nucleatum* Δ*eutV* | Isogenic derivative of CW1 lacking *eutV* | This study |
| *Plasmid* | | |
| pCWU6 | Derivative of pHS30 | (5) |
| pYWC1 | Derivative of pCWU6 | This study |
| pCM-GalK | *C. perfringens* vector expressing *galK* | (5) |
| pΔ*eutN* | pCM-galK derivative; *eutN* deletion vector | This study |
| pΔ*eutLM*$_1$*M*$_2$ | pCM-galK derivative; *eutLM*$_1$*M*$_2$ deletion vector | This study |
| pΔ*eutV* | pCM-galK derivative; *eutV* deletion vector | This study |
| pEutN | Derivative of pYWC1 expressing *eutN* under the control of a RsmA promoter | This study |

**Table S4: Primers used in this study**

| Primer | Sequence[(a)] | Used for |
|---|---|---|
| RT-folK-F | CGGAAGATATAGCAAGGGAAGT | RT-PCR *folK* |
| RT-folK-R | TTCCACAGAAACATCTTTAAGAGC | RT-PCR *folK* |
| RT-folP-F | GCTAATCTTAGATGGAGCAGACA | RT-PCR *folP* |
| RT-folP-R | GCACTACTCTTGATATTTCTTCTTCTG | RT-PCR *folP* |
| RT-eutS-F | AAGAGTATGTGCCAGGGAAAC | RT-PCR *eutS* |
| RT-eutS-R | CATCAAGTCCTAACTTTACACACATATC | RT-PCR *eutS* |
| RT-eutP-F | GCAGATGCTGATATAGAAAGAAGTA | RT-PCR *eutP* |
| RT-eutP-R | CTAAGACTCATTCATTACCAACCTT | RT-PCR *eutP* |
| RT-eutV-F | GGCTATGATGTTGTAGGAGAAG | RT-PCR *eutV* |
| RT-eutV-R | TTAGCAACCTTTAGTCCAGAAA | RT-PCR *eutV* |
| RT-eutW-F | TCTGGTGAAATCGCATATAG | RT-PCR *eutW* |
| RT-eutW-R | CTCTCTGTTGCTCACTATATTCA | RT-PCR *eutW* |
| RT-eutA-F | AAGCAGGAATGACTCCCTCA | RT-PCR *eutA* |
| RT-eutA-R | GAGAAGCTCATAGCTCCTGAACC | RT-PCR *eutA* |
| RT-eutB-F | AGAGTGGCAGCTAAGGTTGT | RT-PCR *eutB* |
| RT-eutB-R | TGATGGTGTAGCTTCTTCGC | RT-PCR *eutB* |
| RT-eutC-F | AGAGTTGGACTTGCAGATGATG | RT-PCR *eutC* |
| RT-eutC-R | TGCTTCAGCTGCTGGTGTACC | RT-PCR *eutC* |
| RT-eutL-F | GAGGTTAAAATGATAAATGATCC | RT-PCR *eutL* |
| RT-eutL-R | TACGCAATTACTACTTCTGCCAT | RT-PCR *eutL* |
| RT-eutM1-F | TGAATTCGAAGGAGATGTAGCAG | RT-PCR $eutM_1$ |
| RT-eutM1-R | TCAGGTCTTGGTATTACATGGC | RT-PCR $eutM_1$ |
| RT-eutM2-F | CAGATGCTATGGTAAAGGCAGC | RT-PCR $eutM_2$ |
| RT-eutM2-R | TCCACTTCTGAGTGAGGTCTTG | RT-PCR $eutM_2$ |
| RT-eutE-F | GTAAGTCCTCACCCAAATGC | RT-PCR *eutE* |
| RT-eutE-R | ACCATTGCTTCTCCACCAGT | RT-PCR *eutE* |
| RT-eutT-F | ACACCATCAGTAAGCAA | RT-PCR *eutT* |
| RT-eutT-R | CCATCAACCATGTCATATATTCT | RT-PCR *eutT* |
| RT-2810-F | GATGAAAACTATATAATTGAATTGG | RT-PCR *2810* |
| RT-2810-R | CATATATTGCATTAGAGATATTGTATAAA | RT-PCR *2810* |
| RT-eutN-F | CTTATAGGTGAAGTTATTGGGAATG | RT-PCR *eutN* |
| RT-eutN-R | TCATATTAAGTATATTTCTTGCTGAGCTTC | RT-PCR *eutN* |
| RT-2820-F | GTAGAAGAACAAATTGGTAGTGG | RT-PCR *2820* |
| RT-2820-R | CTGCTTTTGAATTTGTGTTATTTAC | RT-PCR *2820* |
| RT-eutH-F | GGAATAAATGAAATTATTATCTATATAATGG | RT-PCR *eutH* |
| RT-eutH-R | CCAACTACTGGTCTTAAAATATTTGC | RT-PCR *eutH* |
| RT-eutQ-F | AGAAAAGTAATAAAAGAAGAATTAGG | RT-PCR *eutQ* |
| RT-eutQ-R | CCAGCTCCTAATCTAGGACTTTC | RT-PCR *eutQ* |

| | | |
|---|---|---|
| RT-2835-F | CAGCTGAACTTGCTTGTCTATG | RT-PCR *2835* |
| RT-2835-R | CTCTACTTGATTCTACAATAACATCTCC | RT-PCR *2835* |
| RT-eutG-F | GTCCAATTTGCCTCTTGTATGG | RT-PCR *eutG* |
| RT-eutG-R | GGCTCTTCCATGTGCTATATGA | RT-PCR *eutG* |
| RT-16s-F | TGTCGTGAGATGTTGGGTTAAG | RT-PCR *16s* |
| RT-16s-R | TCCTACTCATCGTAGGCAGTATC | RT-PCR *16s* |
| eutN-up-F | AACTGCAGAGGTATTTAACAGACCAAGGAA | Δ*eutN* |
| eutN-up-R | ACCTATAAGCATATTATCTCCTCTTTA | Δ*eutN* |
| eutV-dn-F | TAATAAGGTGAGTGTATGTCAAAGA | Δ*eutN* |
| eutV-dn-R | CGCGGATCCTATCCTCCCATATCATTTGC | Δ*eutN* |
| $eutLM_1M_2$-up-F | CGCGGATCCCAAGCAGTTACAGAAACAAAAA | $\Delta eutLM_1M_2$ |
| $eutLM_1M_2$-up-R | TTCCACTTCTGAGTGAGGTCATTCTTCTGCCATTTTAGCA | $\Delta eutLM_1M_2$ |
| $eutLM_1M_2$-dn-F | TGCTAAAATGGCAGAAGAATGACCTCACTCAGAAGTGGAA | $\Delta eutLM_1M_2$ |
| $eutLM_1M_2$-dn-R | CGGGGTACCAATAAACCTGCCTAGTTTCTCA | $\Delta eutLM_1M_2$ |
| eutV-up-F | AGAGGAGCTCGATTTACCTTTTGATGAAAGAG | Δ*eutV* |
| eutV-up-R | AGAGGGTACCCTTGTAAGTGTTTCATCTTCCAC | Δ*eutV* |
| eutV-dn-F | AGAGGGTACCGAAAGAGCCAAAGGAATAGTTATG | Δ*eutV* |
| eutV-dn-R | AGAGGTCGACCTAAGTAAACTTGCCACTGTCTG | Δ*eutV* |
| com-eutN-F | CGCGGATCCATGCTTATAGGTGAAGTTATTGG | pEutN |
| com-eutN-R | CGGGGTACCCTTTGACATACACTCACCTTATTATT | pEutN |

[a] Underlined are restriction site sequences.

**References**


1. Thompson JD, Higgins DG, Gibson TJ. 1994. CLUSTAL W: improving the sensitivity of progressive multiple sequence alignment through sequence weighting, position-specific gap penalties and weight matrix choice. Nucleic Acids Res 22:4673-80.

2. Thompson JD, Gibson TJ, Plewniak F, Jeanmougin F, Higgins DG. 1997. The CLUSTAL_X windows interface: flexible strategies for multiple sequence alignment aided by quality analysis tools. Nucleic Acids Res 25:4876-82.

3. Varadi M, Bertoni D, Magana P, Paramval U, Pidruchna I, Radhakrishnan M, Tsenkov M, Nair S, Mirdita M, Yeo J, Kovalevskiy O, Tunyasuvunakool K, Laydon A, Zidek A, Tomlinson H, Hariharan D, Abrahamson J, Green T, Jumper J, Birney E, Steinegger M, Hassabis D, Velankar S. 2024. AlphaFold Protein Structure Database in 2024: providing structure coverage for over 214 million protein sequences. Nucleic Acids Res 52:D368-D375.

4. Takenoya M, Nikolakakis K, Sagermann M. 2010. Crystallographic insights into the pore structures and mechanisms of the EutL and EutM shell proteins of the ethanolamine-utilizing microcompartment of *Escherichia coli*. J Bacteriol 192:6056-63.

5. Wu C, Al Mamun AAM, Luong TT, Hu B, Gu J, Lee JH, D'Amore M, Das A, Ton-That H. 2018. Forward Genetic Dissection of Biofilm Development by *Fusobacterium nucleatum*: Novel Functions of Cell Division Proteins FtsX and EnvC. mBio 9.

**A**

```
EutM1       ---MKALGLIETKGMVGAIVAADIALKTAQVELINKECVKGGLVCIEFEGDVAAVKASVEAAVTAIKDMGIYVGS     72
EutM2       MSTLNALGMIETKGLVAAVEAADAMVKAANVTLVGKELVGGGLVTVMVRGDVGAVKAATDAGAAAADRVGELISV     75
               ::***:*****:*.*: ***  :*:*:* *:.** * **** : ..***.****:.:*..:* . :*  :.

EutM1       HVIPRPDDSVEKIIKRKNETSKEEVIEEKVEKIKKETKDIEEEIEEINEILKVSKNKKQKNKK 135
EutM2       HVIPRPHSEVELILPKSNN-------------------------------------------- 94
            ******...** *: :.*:
```

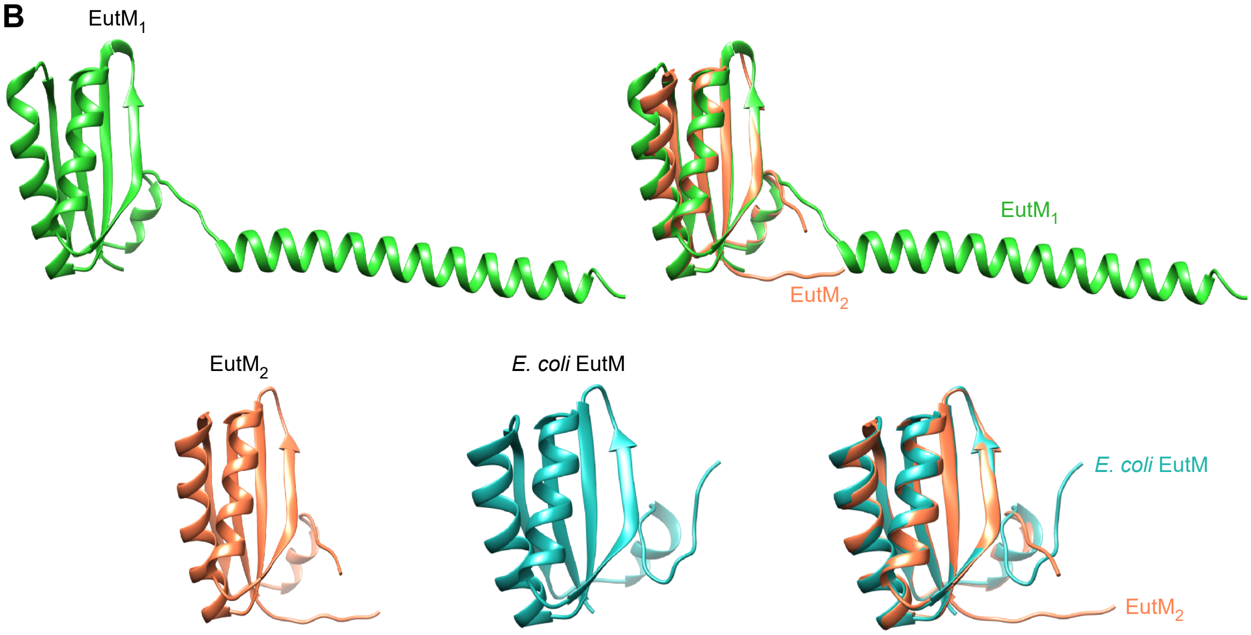


Figure S1: Franklin et al.

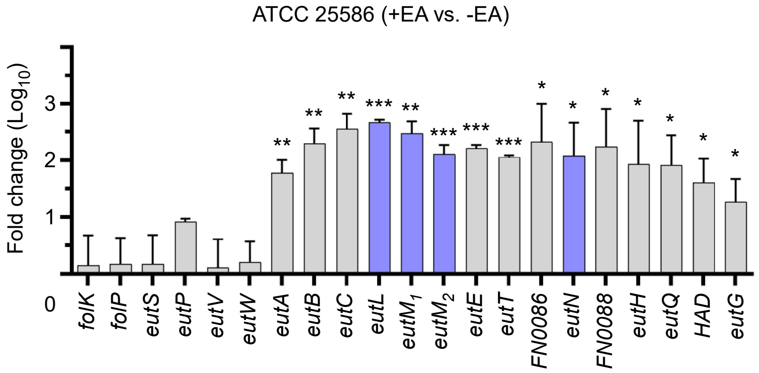


Figure S2: Franklin et al.

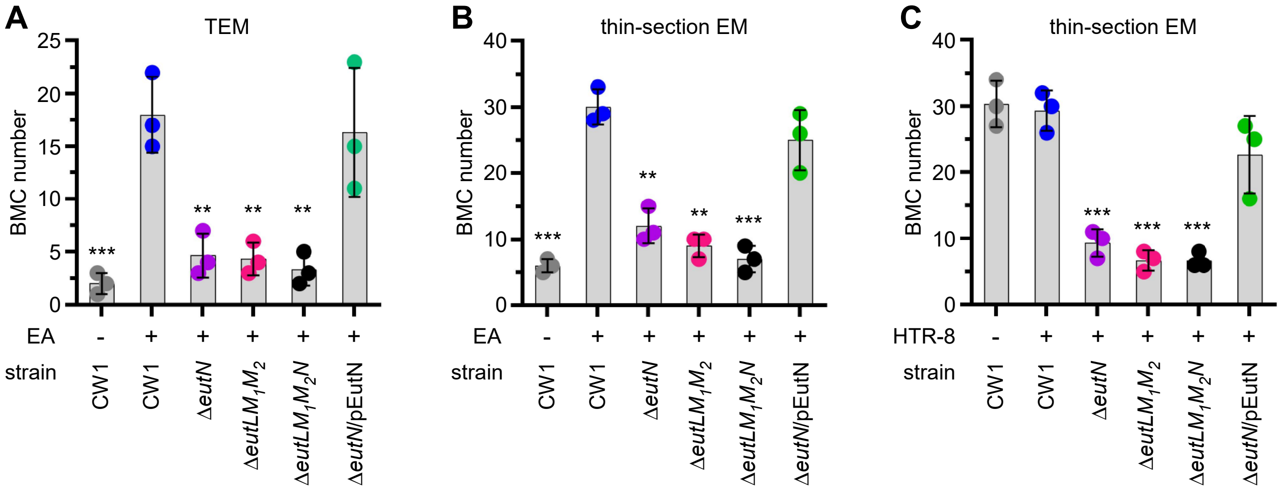


Figure S3: Franklin et al.
